# Implementing voluntary counselling and testing services among university students in Mozambique: a mixed methods process evaluation

**DOI:** 10.3389/fpubh.2026.1911945

**Published:** 2026-08-24

**Authors:** Arlinda Basílio Zango, Sarah E. Stutterheim, Rik Crutzen

**Affiliations:** 1 Departamento de Investigação em Saúde e Bem-Estar, Faculdade de Ciências de Saúde, Universidade Católica de Moçambique, Beira, Mozambique; 2 Department of Health Promotion, Care and Public Health Research Institute, Faculty of Health, Medicine and Life Sciences, Maastricht University, Maastricht, Netherlands

**Keywords:** HIV, implementation, students, testing, university, voluntary counseling

## Abstract

**Introduction:**

Understanding the relationship between the program implementation and its context is a key strategy for explaining why a program produces its effects and for informing decisions about its adaptation. We evaluated the implementation of a program promoting the use of Voluntary Counseling and Testing (VCT) among university students.

**Methods:**

The study was conducted at the *Universidade Católica de Moçambique* using a mixed-methods approach to examine context, reach, dose, and fidelity. Data collection involved observation, interviews, and focus group discussion (FGDs). The study participants included teachers, peer activists, health advisors, and program users. Peer activists were eligible if they were actively engaged in sensitization activities. Health advisors and life skills teachers were required to hold a valid full-time employment contract with UCM. Users were eligible if they were first-year student aged 16 to 25 years. Quantitative data were analyzed using descriptive statistics. FGD data were analyzed using thematic analysis.

**Results:**

The implementation context was shaped by compliance with preventive measures against the COVID-19 pandemic. Attendance at life skills lessons was high, with more than half of users reporting attendance at 70–100% of scheduled lessons. Attendance at sensitization sessions was also acceptable, with nearly half of users reporting attendance at 70–100% of scheduled sessions. A notable number of participants were not reached by VCT, and implementation fidelity was not fully maintained across all program components. Users indicated that initial exposure to the program elicited emotional responses; however, over time, they normalized the topic and became more comfortable discussing sexuality. Persistent challenges hindered engagement with VCT. Consequently, users recommend expanding the program and making it more inclusive; improving the overall delivery; and reviewing the timing, duration, frequency, as well as diversifying the content of awareness.

**Conclusion:**

The results of this study contribute meaningful and updated evidence on the implementation of VCT promotion programs at university in resource-limited settings. These findings may guide the future development, implementation, and adaptation of VCT promotion programs in similar contexts. We therefore recommend that the future implementation be informed by evidence and recommendations drawn from program users.

## Introduction

Process evaluations are a critical component of evaluating health programs, particularly in the field of behavior change. They provide valuable insights into factors that influence program effectiveness and help stakeholders understand whether, how, and why a program was delivered as planned (1–3). Essentially, process evaluations focus on understanding the relationship between program implementation and its context and explaining why a program produces positive or negative outcomes. Process evaluations also serve to inform decisions about the extent to which a program can be adapted to other settings (3, 4). Process evaluations can address multiple domains, including context, reach, dose, responsiveness, and fidelity (1). This study aimed to evaluate the implementation of a program promoting the use of Voluntary Counseling and Testing (VCT) services among university students at the Catholic University of Mozambique (UCM) in Beira, central Mozambique.

VCT is a global strategic approach proposed by the World Health Organization (WHO) and the Joint United Nations Programme on HIV/AIDS (UNAIDS) in 1994 to prevent and control HIV and related health conditions, including STIs, opportunistic infections, and the provision of psychological support (5). In the Mozambican context, VCT is applied to enable the general population to know their health status regarding various health conditions, including STIs, with particular emphasis on the diagnosis of HIV infection (6).

VCT is implemented as both a primary and a secondary prevention strategy (5) and comprises both pre- and post-test counseling. As a primary prevention measure, VCT supports sexual health by providing individuals with information and skills to support informed decision-making, promote protective sexual behaviors, and reduce the likelihood of acquiring sexually transmitted infections (STIs), including HIV. As a secondary prevention measure, VCT facilitates early detection, linkage to care, early treatment, and appropriate care for people with HIV, thereby preventing disease progression and reducing transmission rates (5–7).

The Joint United Nations Programme on HIV/AIDS (UNAIDS) identifies adolescents and young adults aged 15–24 years, an age range that includes most university students, as a key population bearing a substantial burden of STIs, including HIV (8). High prevalence and incidence of STIs and HIV have been reported in this age group across countries (9, 10), including Mozambique (11–14). Consequently, promoting sexual and reproductive health (SRH) services among adolescents and young adults, including university students, is considered strategic in public health. In line with this, the Catholic University of Mozambique (UCM) promotes SRH, including the use of VCT services, through a structured behavior change program.

The program consists of three components: (1) life-skills-based curricular lessons for first-year students; (2) awareness activities addressing sexuality and STI/HIV prevention; and (3) provision of VCT services. The implementation of such a program within university setting represents a novel approach in Mozambique. Therefore, assessing its implementation context, including factors that may influence context, reach, dose delivered and received, fidelity, responsiveness, and recruitment is relevant.

Previous studies on implementation of STI and HIV prevention programs have yielded valuable findings. For instance, a study conducted in Zambia reported that key contextual factors affecting implementation included funding fragmentation, absence of monitoring systems in schools, limited community engagement, sociocultural barriers, insufficient teaching competencies, and inadequate time allocation (15). In a South African study, fidelity was negatively affected by the restrictive public health measures imposed during the COVID-19 pandemic. Program reach was similarly compromised, as the peer-led component failed to improve uptake of diverse HIV prevention services, including VCT and linkage to care (16).

Additionally, in another South African study, participants were generally comfortable sharing sexual health concerns with same-age peers. However, some participants demonstrated limited interest in the broader sexual health information delivered, while others were uncomfortable receiving information from non-professional peers (17). Lastly, in a related study, the majority of participants received the intervention with low to medium fidelity (18).

Notably, there is a considerable body of published literature reporting on process evaluations that examine context, reach, fidelity, and other implementation outcomes. This study seeks to contribute to that evidence base by providing updated information that can then guide policymakers and public health practitioners, on how to design, improve, and implement STI prevention programs, including VCT promotion tailored to the needs of university students, in low resource settings. Specifically, this study aimed to evaluate the implementation of a program promoting the use of Voluntary Counseling and Testing (VCT) services among university students at the Catholic University of Mozambique (UCM) in Beira, central Mozambique: (1) describing program delivery in terms of context, reach, dose delivered and received, and fidelity, and (2) exploring the experiences and barriers encountered by students in adopting recommended behaviors.

## Materials and methods

### Study design

This study was a process evaluation conducted as part of a larger project evaluating an STI prevention program among university students in Mozambique (19). In this paper, we present the findings concerning the implementation process following Standards for Reporting Qualitative Research SRQR (20).

Our process evaluation study utilized a mixed-methods approach, combining qualitative and quantitative elements to examine multiple implementation aspects: context, reach, dose delivered, dose received and fidelity. The process evaluation summarized in Table 1 involved reviewing the program’s documents and records, conducting individual interviews with program implementers and student cohort members (i.e., intervention users). We also conducted four focus group discussions with users (FGD).

**Table 1:** Process evaluation overview.

Domain	Definition	Questions/purpose	Indicators	Methods
Context	External aspects of the program that influenced the implementation of one or all components of the program	Has the context changed for STI/HIV prevention programme during the intervention implementation? What changes have been made in curricular lessons provision? What changes have been made in awareness activities delivery? What changes have been made in STI/HIV screening offering services?	Classroom conditions for group discussion activities, design/schemes for group activities. Students’ criterion to attend life skills lessons. Training certificates of the implementers. Standard Operations Procedures to delivery of sensitizing activities. Guideline for STI/HIV screening and Standard Operation Procedures.	Observation of classroom lessons and handbooks. UCM guideline. Observation, and interview of selected sample of users Interview of selected sample of users.
Reach	Proportion of users engaging and patriating in each program components	To what extent is the intervention reaching anticipated participants? Are unanticipated groups taking part? Whom are these groups? Are any groups being missed?	Percentage of university students who use STI/HIV screening services, distributed by year or class. Percentage of users who are not university students.	Client records during research period at the STI/HIV counselling office. Participation rates of students in the life skills classes.
Dose delivered	Extend to which each of the three program components are delivered	How much of the programme components are being delivered? What is being omitted or delivered inconsistently? Why?	Percentage of lessons provided, and average of time length of each scheduled lesson. Percentage of sensitizing sessions delivered, and average of time length for each session. Percentage of screening services offered and average of time length for each client attended.	Book-lessons record, and checklist or time record for lessons deleverage. Records of sensitizing activities and time record sheet. Checklists and time record sheet.
Dose received	Extend to which each program components are received	What is the average dose received by intervention (participants for each component)? What parts of the intervention components are not received consistently? Why?	Percentage of 1st year students attending lessons at each scheduled time. Percentage of intended participants attending each session of sensitizing activity. Percentage of university students who seek STI/HIV screening service per month over a year	Attendance record sheets for each class and scheduled lesson. Analysis of attendance record for each session of sensitizing activities Review and analysis of counselling records. Interview to selected sample of users
Fidelity	Extend to which program components are delivered as planed	Have the theory and evidence-based change methods been appropriately implemented in the intervention application?	Degree to which activities of each component are linked to the theoretical methods and practical application to specific determinants.	Interview to selected sample of users.

Adapted from: Zango et al., (19).

### Study site

The study was conducted in two faculties, the Faculty of Health Sciences (FHS) and in the Faculty of Economics and Management (FEM) at the *Universidade Católica de Moçambique* (UCM) in Beira, Sofala province, central Mozambique. Mozambique is one of the low-income countries in Southern Africa, with a 2,700 km coastline along the Indian Ocean. The country has a population of approximately 34.09 million people, of whom approximately 21.6% are aged between 15 and 24 years. UCM is a private university with faculties across the southern, central, and northern regions of Mozambique. In Beira, UCM comprises two faculties, the Faculty of Health Sciences and the Faculty of Economics and Management, and one e-learning institute. In both faculties, students receive social and health support from a priest, social assistants, and health advisors. UCM was selected through convenience sampling because it is the only university in the region offering a formal and structured sexual and reproductive health program comprising curricular lessons, awareness activities, and the on-site provision of VCT services.

### Description of the STI prevention program

This STI prevention program comprised three main components: (a) life skills education for first-year students, (b) awareness activities focused on sexuality and STI/HIV prevention, and (c) provision of VCT services within university faculties. The implementation team comprised two health advisors, six life skills teachers, and 32 peer activists.

#### Life skills education

The life skills curriculum followed a student-centered learning approach, supported by complementary teacher and student handbooks as core didactic materials. The course comprised 64 total hours, divided between 34 h of lectures and 30 h of practical sessions. Sexual and reproductive health (SRH) topics constituted approximately 50% of the content, including 16 h specifically dedicated to STIs and HIV education. For research purposes, the behavioral aspects were addressed through eight two-hour sessions, delivered twice weekly during the first academic semester.

#### Awareness activities

Peer activists conducted four awareness sessions that aligned with the life skills module structure and sequence. Each session focused on specific health topics, including the progression of HIV infection, STI symptoms, help-seeking processes, complications of untreated STIs, and treatment protocols for STIs and HIV. The peer activists team at the FHS is called PABODZY and at the FEM is called TIRITESSE.

#### Voluntary counseling and testing

Health advisors provided STI and HIV counseling and testing services in private rooms at each faculty location. Service delivery followed the national guidelines (21), which outline three sequential counseling steps: (a) pre-test counseling, (b) counseling during test/examination, and (c) post-test counseling.

During the intervention and study period (May 2021 to June 2022), the world was impacted by the COVID-19 pandemic. This pandemic necessitated changes in the implementation of all three intervention components due to restrictions on face-to-face activities at schools and universities.

### Study participants

Study participants included life skills teachers, peer activists, health advisors, and first-year student cohort participants. All life skills teachers, health advisors, and a sample of peer activists were invited to participate in the study. Additionally, a sample of first-year students was systematically selected from the cohort using stratified random sampling, with stratification based on faculty affiliation and gender. The sampling process continued until data saturation was reached, which was expected to occur after 6 to 12 interviews per gender per faculty, based on established guidelines (22–24). The same sampling strategy was applied to select participants from the pool of peer activists. Specifically, from the list of students enrolled at the Faculty of Health Sciences and the Faculty of Economics and Management, participants were randomly selected within gender groups and invited to take part in the focus group discussions until the required sample size was reached (four groups of 6 to 12 participants per group).

### Eligibility criteria

Peers activists were eligible if they were actively engaged in sensitization activities and enrolled in years two through four of standard programs, or years one through six of the medicine program at UCM. Health advisors were required to have a valid full-time employment contract with UCM and formal assignment to provide voluntary counseling and testing services. Life skills teachers needed both a valid UCM employment contract and formal assignment to teach life skills during the 2021 academic year. Users were eligible if they were first-year UCM students aged 16–25 years.

### Data collection

The comprehensive process evaluation methodology is detailed in the protocol (19), and summarized in Table 1. “The interview guides and questionnaires were developed in English by the research team, based on the specific aims of the study, and subsequently translated into Portuguese, the official language of Mozambique. The tools were developed specifically for this study, drawing on the relevant theoretical framework and the existing literature on process evaluation. To assess content validity, the tools were reviewed by all members of the research team and refined through discussion and consensus. The tools were then pilot-tested with a small group of participants, and the language was subsequently refined based on the feedback obtained. The final versions of the tools are available as appendices for each data collection technique in the supplementary material, and can also be accessed at: https://www.frontiersin.org/articles/10.3389/frph.2021.745309/full#supplementary-material.

Data collection involved multiple methods: (a) Direct observation of classroom activities; (b) Individual semi-structured interviews; (c) Focus group discussion (FGD); (d) Monitoring of VCT service utilization. First, we interviewed life skills teachers using a guide (Appendix PE1) that included four closed-ended demographic questions and five open-ended questions exploring their opinions, experiences, and motivations in teaching life skills to university students.

Second, we interviewed health advisors using a structured guide (Appendix PE2) comprising four closed-ended demographic questions and five open-ended questions. These questions assessed their experiences providing VCT services at the university, perceptions of program impact on students, implementation challenges, and suggestions for program improvement.

Third, we conducted interviews with peer activists using a structured guide (Appendix PE3) containing eight closed-ended and five open-ended questions. These questions explored activists’ engagement in sensitization activities, their perspectives on the activities’ importance to the users, and recommendations for program enhancement. Qualitative data from implementers were not reported here.

Finally, we conducted interviews using a structured guide (Appendix PE4a; b; c) and FGDs with program users using a guide (Appendix PE5) containing ten open-ended questions. These questions explored participants’ feelings, experiences, and suggestions for program improvement. Four FGDs were conducted: two female-only and two male-only student groups.

All FGDs were audio-recorded using a smartphone and manually transcribed verbatim for analysis. The transcriptions were assigned unique identification codes. Two members of the research team (the transcriber and the principal investigator) reviewed the transcriptions for accuracy.

## Data analysis

We analyzed quantitative data through descriptive statistics and proportions. We applied thematic analysis to data of the FGDs (25–28). The thematic analysis consisted of six steps (25, 27, 29). First, we familiarized ourselves with the content of the data and identified the meanings by reading and rereading the interview transcriptions making notes on both the entire dataset and individual transcripts. Second, we identified and defined labels for data features relevant to our research question by systematically analyzing the data through coding. Third, we reviewed the codes and identified areas of similarities and overlapping between them. We categorized codes that shared unifying features and constructed thematic maps to organize the categories into specific themes. Fourth, we conducted quality checking and developed the thematic maps through a recursive process. Once we confirmed that our thematic map accurately reflected the coded data, we evaluated the themes in relation to the entire dataset. Fifth, we refined, defined, and named the themes by selecting extracts to present and analyze, then crafting a coherent narrative around these extracts. We combined data extracts to illustrate claims about the dataset, supplementing them with analytical commentary on specific excerpts. Finally, we produced a report that provides a compelling narrative. The findings were interpreted through a thematic representation of users’ experiences, beliefs, and other relevant aspects following exposed to the program.

The study received ethical approval from the *Comité Interinstitucional para Bioética de Sofala* (CIBS) under registration number 004/CIBS/2020 and obtained local administrative authorization. Prior to enrollment, all participants provided written informed consent.

## Techniques to enhance trustworthiness

To enhance four criteria of trustworthiness (i.e., credibility, transferability, dependability, and confirmability); (27, 30, 31), we followed: (a) Methodological triangulation: collecting data from different participants using multiple techniques; (b) Prolonged engagement: spending sufficient time in the context to build trust and gain deeper insights into participants’ experiences, behaviors, and beliefs; (c) Reflexivity: acknowledging personal biases and preconceptions throughout the research process; (d) Thick description of the research context, including participants and methods; (e) Comprehensive sampling strategy description, detailing participant selection criteria; (f) Methodological documentation; (g) Audit trail: recording all decisions made during the study; and (h) Peer debriefing: engaging with expert co-authors on review and interpretation of findings to minimize personal bias.

Especial attention was paid regarding reflexivity: All members of the research team (AZ, a PhD candidate; RC, a cognitive psychologist with research expertise in health behavior change; and SS, an interdisciplinary mixed-methods researcher with expertise in sexual health and HIV) acknowledge that the findings of this study may have been influenced. For instance, AZ may have been less critical and impartial in her interpretation of the data, given that she is an early-career researcher in this field and lives in the same resource-limited context as the participants. While, RC and SS, who come from entirely different contexts, may have brought greater impartiality to the interpretation of the data. We maintained a self-critical stance and engaged in systematic discussions at each stage of the research process, from interview transcription coding and theme development to the narrative description of the results, reflecting on how our individual expertise, experiences, and beliefs might have influenced the interpretation of the qualitative data.

## Results

### Participants characteristics

We interviewed six teachers: four females and two males. The mean age among teachers was 35 years (SD = 6) for females and 32 years (SD = 4) for males. Three of the teachers were nurses and the others were from different professions (one lawyer, one marketing and public relations professional, and one Portuguese teacher). On average, they had been teaching life skills education for about 3.5 years (SD = 1.87). We also interviewed 18 peer activists, of whom 12 (67%) were female. The mean age among females was 21 years (SD = 1.2), and among males was 25 years (SD = 3.3). Only one health advisor responded to our questionnaire. Additionally, we included 39 students from the intervention arm, of whom 56% (22/39) were female. The mean age was 19 years (SD = 0.7) among female students and 19 years (SD = 1.1) among male students.

### Quantitative data

Most of peer activists 14 (78%) were invited to join and engage in provision of awareness activities by a friend or by a member of the team, while only 4 (22%) joined the team voluntarily. Almost all peer activists perceived that engagement in awareness activities was important for themselves as well as for the user students. Peer activists also affirmed that STI awareness activities for students were a priority and that they were committed with promoting prevention of STIs and HIV.

Table 2 summarizes the characteristics of program users (n = 39) and presents their estimations on program reach, responsiveness, and fidelity. In terms of reach in the life skills education, 59% of participants reported attending all of the scheduled life skills lessons, while 20% of participants attended 90, and 21% of participants reported 70% or less attendance. Regarding fidelity, 52% of participants indicated that each topic of every lesson was discussed, compared to 48% of the respondents who reported that not all topics were discussed. However, only 2% of participants reported discussion of topics before lectures. The majority of users (85%) affirmed that teachers mostly provided a summary for each topic, and only 50% declared that these summaries were given at the end of lectures.

**Table 2:** Characteristics of users and their assessments of program reach, responsiveness, and fidelity.

Variables	*N*	Percentage (%)
Sex	*n* = 39	
Female	22	56.4
Male	17	43.6
Age (mean/SD)		
Female	18.73(0.730)	
Male	19.29(1.047)	
Life skills education		
Lessons attendance	*n* = 39	
Every time (100%)	23	59.0
Usually (90%)	8	20.5
Frequently (70%)	4	10.3
Never	4	10.3
Discussion of a topic on life skill subject	*n* = 35	
Every time	3	8.6
Almost every lesson	15	42.9
Sometimes	12	34.3
Almost never	5	14.28
Discussion moment	*n* = 34	
After lecture	17	50.0
During lecture	10	29.4
Before lecture	7	20.6
Provision of the summary of a topic	*n* = 35	
Every lesson	16	45.7
Almost every lesson	13	37.1
Sometimes or never	6	17.14
Summary moment	*n* = 34	
At the beginning of the lecture	8	23.5
During lecture	6	17.6
At the end of a lecture	10	29.4
After discussion with students	7	20.6
Do not remember	3	8.8
Awareness activities		
Attendance to awareness activities	*n* = 39	
Every time (100%)	5	12.8
Almost every time (90%)	4	10.3
Occasionally (70%)	19	48.7
Almost never or never	11	28.21
Theater presentation	*n* = 32	
Yes	13	40.63
No	19	59.37
Video presentation	*n* = 32	
Yes	6	18.75
No	26	81.25
Questions during awareness session	*n* = 32	
Yes	11	34.37
No	21	65.63
Discussion during awareness activities	*n* = 32	
Yes	9	28.12
No	23	71.18
Provision of a summary of benefits of the activities	*n* = 32	
Yes	8	25.0
No	24	75.0
Additional awareness activities	*n* = 32	
Yes	3	9.37
No	29	90.63
VCT services provided		
Frequency of use VCT services	*n* = 39	
Never	19	48.7
Once a year	11	28.2
Twice a year	3	7.7
Three times a year	3	7.7
Four or more times a year	3	7.7
Discussed STI testing process	*n* = 20	
Yes	9	45.0
No	11	55.0
Discussed testing result	*n* = 20	
Yes	8	40.0
No	12	60.0
Discussed testing calendar	*n* = 20	
Yes	5	25.0
No	15	75.0
Discussed other issues	*n* = 20	
Yes	4	20.0
No	16	80.0
User responsibility at testing meeting	*n* = 20	
Somewhat responsible	9	45.0
Mostly or completely responsible	11	55.0
Consider the counseling process as collaborative	*n* = 20	
Might not consider	5	25.0
Definitely consider	15	75.0

Regarding reach for awareness activities, 23% of participants reported attendance of 90 to 100%, while 28% referred attendance of less than 70%. In terms of fidelity, 41% of participants affirmed presentation of a short theater piece in awareness activities, 19% reported video presentation, and 34% reported starting with questions for discussion at each session, only 28% of participants indicated that all topics in awareness activities were discussed, and 25% of respondents reported provision of summaries on the benefits of discussed topics.

In terms of reach for VCT service, of the 39 participants, 51% reported having visited a VCT service for counselling and testing on STIs and HIV during the study period. Regarding fidelity on VCT services, 45% (9/20) of participants affirmed having discussed the STI testing process, 40% (8/20) confirmed that they discussed testing results with the health advisor, and 25% (5/20) affirmed having discussed the STI/HIV testing calendar. Furthermore, 55% (11/20) affirmed that they were mostly or completely responsible for the discussion points during their visits to the health advisor, and 75% (15/20) considered the counselling process to be collaborative.

### Qualitative findings

#### Contextual situation

Observational findings revealed that the primary contextual factors affecting program delivery were related to adaptations made to comply with recommended measures to prevent the spread of COVID-19. Consequently, some life skills lectures were adapted and delivered through online education platforms, including Zoom, Google Meet, and Microsoft Teams. As a result, some students may have missed lessons due to not owning a smartphone or personal computer, while others experienced disruptions caused by limited data or unstable internet connectivity.

Sensitization activities were planned to be delivered using a face-to-face approach, involving interpersonal interaction and discussion. Nevertheless, some activities were postponed or shortened even during periods when in-person educational activities were permitted. Peer activists were pressed for time and compelled to condense some sessions, often limiting opportunities for attendees to ask questions, as they prioritized delivering the core content of each planned topic.

Regarding provision of VCT services, users were encouraged to seek this services at any available venue, as well as at the faculty during periods when face-to-face service provision was permitted. As a result, tracking student engagement with VCT services and recording testing outcomes proved difficult. Furthermore, some students may have sought counselling and testing from health advisors who were not part of the program’s trained personnel, making it difficult to accurately monitor service utilization.

Additionally, health advisors highlighted the need for refresher training in STI and HIV counselling and testing, as their previous training had not been updated for an extended period. This refresher training was intended to be conducted by a team from the Department of Public Health of the Provincial Directorate of Health. However, it was not carried out, as attention within health services had been redirected toward managing the COVID-19 pandemic. It was also noted that not all life skills teachers had a background in health sciences or biomedical fields.

### Focus group discussion findings

Analysis of the data from the focus group discussion (FGDs) yielded three overarching themes: (1) Overall impressions of the program, in which participants indicated emotional reactions, normalized sexual and reproductive health topics over time, and found the program interesting; (2) Perceived impact of the program, encompassing increased knowledge and awareness, and strengthened self-efficacy, empowerment and skills, and the perceived impact on actual VCT service use; and (3) Recommendation for improvement and future implementation, in which users indicated the need for expanding and making the program more inclusive, improving the delivery of the program, adjusting timing, duration and frequency, diversifying the program, and maintaining the program.

### Overall impressions of the program

#### Emotional reactions

Program users reported having experienced different emotions as a result of participating in the sexual and reproductive health (SRH) program focusing on voluntary counseling and testing (VCT) promotion. The emotional responses were characterized by mixed feelings, including fear and curiosity, surprise and embarrassment, and worry and uncertainty. As stated by a male student from the Faculty of Health Sciences (FHS): “I was kind of scared, but also curious at the same time” (B-FHS). Students expressed curiosity about what the program would convey and anticipated acquiring knowledge and skills related to sexual health. Users further indicated that they had not expected the Catholic University to implement a program addressing SRH, given that this topic remains a taboo in Mozambique. The subject is typically discussed in informal settings; therefore, having it addressed in a formal academic context was perceived as a valuable experience from which students expected to gain considerably, as affirmed a male student from the FHS.

“When I initially saw this program being introduced at the university, I first felt surprised. I did not expect that at this university we would address topics related to sexual and reproductive health, so at first I felt a bit embarrassed” (B-FHS).

#### Sexual and reproductive health is a normal topic

SRH programs delivered in school settings, including those facilitated by the Catholic Church, were not entirely new to all program users. Some students reported that they were neither surprised nor embarrassed, as they had previously been exposed to and had attended similar programs at the secondary school level. However, the behavior change methods and their practical applications used to deliver the content at the university were perceived as more varied and professionally structured. Users indicated that they were pleased and felt privileged to continue engaging with SRH promotion activities at the university level, and expressed an expectation to further deepen their knowledge of sexuality, including the prevention of STIs, including and HIV, as described a female student from the FHS.

“I’ve always had similar programs at the schools where I studied, and I participated in many similar activities. So for me, it’s an honor to continue with this, because I’ve always liked learning and I’ve always liked taking part in similar programs. For that reason, it’s always good to be here; as we have meetings, I keep learning more and more” (G-FHS).

#### Interesting program

Program users described the promotion of the SRH program at the university as an interesting and effective approach, reflecting that some students have positive attitude toward some or all program components. Students noted, for instance, that peer activists presented engaging topics in a friendly and approachable manner. As stated by a male student from the FHS: “They came as friends, to share, to show, and to present something, and also to encourage questions” (B-FHS). Users further described the sensitization sessions as constructive debates on sexuality and other relevant life skills topics. They reported that sensitization sessions delivered through theatrical presentations had a strong impact, as these motivated attendees to reflect on and engage in healthy sexual behaviors. Students who attended the theatrical presentation sessions felt a personal connection with the message conveyed, affirming that it was directly relevant to their daily lives with regard to sexuality and SRH behavior, as stated by a male student from the FHS.

“I really liked the presence of the young people from the PABODZI group, because first of all, seeing young people who are our peers presenting theatrical plays that truly portrayed what we do in our daily lives was very gratifying” (B-FHS).

### Perceived impact of the program

#### Impact on personal determinants of VCT: increased knowledge and awareness

Program users expressed having benefited considerably from the program, reporting that it broadened their understanding and led them to reconsider their perspectives on sexuality and prevention of STIs, including HIV, make more informed decisions, and reflect more critically on their SRH behavior. Users affirmed that the program was educational, enhanced their knowledge, and guided students to seek assistance from a healthcare provider when faced with health concerns. At the same time, they acknowledged that initially they had perceived the life skills subject as unrelated to their degree program. As stated by a male student from the Faculty of Economics and Management (FEM): “At first I thought, well, it’s just another subject added to increase the number, like many others, meaning it’s not directly related to my degree” (B-FEM). However, by the end of the program, participants came to recognize the life skills subject as a valuable source of knowledge applicable to their lives, as expressed by a female student from the same faculty: “it brought wisdom to me about things that I did not know before” (G-FEM). Program users reported that, following exposure to the program, they developed a heightened awareness of their own behaviors and decision-making processes, as expressed by a female student from the FEM: “people were able to understand, or to take time to reflect, to analyze what to do and how to behave” (G-FEM).

Although most program users perceived impact of the program on their knowledge about STI, including HIV prevention, knowledge gaps and limited understanding of the benefits and purposes of VCT services were not fully addressed among all users. Some users indicated that they had insufficient information regarding the availability of VCT facilities at the faculty or at nearby locations providing adolescent and young adult health services.

Fewer students also reported a lack of clarity regarding the reasons for seeking VCT services, as stated by a male student from the FEM: “it is extremely important for people to know the real reasons for following the schedule” (B-FEM). This suggests that some participants remained insufficiently informed about the scope of VCT services in the Mozambican context, which are intended not only to address specific health concerns but also to equip individuals with the knowledge and skills necessary to make informed decisions regarding their sexuality, including those who are not yet sexually active. A prevailing misconception identified among students was the assumption that VCT services are exclusively intended for sexually active individuals concerned about STIs and HIV as affirmed a male student from the FHS.

“So I think that most of us—for example, I do not go because of that way of thinking, like, I have not done anything that would require me to get tested” (B-FHS).

Furthermore, some program users revealed a lack of information regarding VCT services, particularly with respect to the recommended frequency of regular visits to a healthcare advisor, as indicated by a male student from the FEM: “we did not even realize that we had to visit the counseling office” (B-FEM). Users perceived that a visit to a healthcare provider for counseling and screening was only warranted when they were concerned about or presenting with symptoms of an STI.

#### Impact on personal determinants of VCT: increased self-efficacy, empowerment, and skills

Following exposure to the program, users reported a reduction in shame, increased confidence in their ability to seek and use VCT services, and greater readiness to accept the results of STI and HIV testing. They further indicated that, as a result of the encouragement and advice received during counseling and testing sessions, as well as guidance provided by peer activists during sensitization sessions, they had come to normalize the practice of getting tested regularly, as indicated a male student from the FHS.

“…I was very afraid, I do not know what came over me that day, but I had that courage. I went there and did the test,” (B-FHS).

The program further provided a sense of empowerment and strengthened self-efficacy to students particularly women, as illustrated by a female student from the FHS: “We can now know how to speak and express our ideas” (G-FHS). Female participants also developed skills and the ability to articulate their feelings and opinions on relationship with partners, as further stated by the same participant: “…and our ability to have a voice” (G-FHS). Users stated that women are traditionally perceived as submissive in their social and relational contexts; however, following engagement with the negotiation skills component of the program, they reported having gained confidence, the ability to negotiate personal ideas, and the capacity to make autonomous decisions regarding acceptance or refusal of sexual advances. Participants reported having acquired and developed skills in negotiation, persuasion, and active listening, recognizing these as key competencies for success across various domains of life, including sexuality and the prevention of STIs and HIV. At the same time, they acknowledged that negotiation does not always yield positive outcomes and that the process may sometimes fail, leading to unfavorable results, as stated a female student from the FHS.

“I think it helps us broaden the way we see things, because, as she said, there was a lot of submissiveness on the part of women in society. If a man said no, it was no—there was no further discussion. But now, after the lectures, we can expand our knowledge and our ability to have a voice. We can now know how to speak and express our ideas, how to talk, and then we end up negotiating and being able to achieve positive results” (G-FHS).

#### Perceived impact on VCT service use

With regard to the impact of the program on VCT service utilization, some users expressed that they felt motivated and engaged with VCT services following attendance at sensitization activities held at the faculty. This was illustrated by a male student from the FEM: “Yes, going to do the test happened on a day when we had a talk there in the Beira hall” (B-FEM). Students reported having had the opportunity to visit a healthcare advisor even in the absence of a specific health concern, a decision they attributed to having been informed about the role of VCT services and the different modes of HIV transmission during life skills lectures and sensitization sessions, as reported by a female student from the FHS (G-FHS).

“…but last year we had a lecture where they told us that it’s not only through sexual relations; there are other ways of getting HIV. After that, I did the test…” (G-FHS).

While some users demonstrated an understanding of, and appreciation for, the benefits of VCT services, certain barriers were reported as impediments to their regular engagement with such services. Some users noted, for instance, that they experienced a lack of motivation when considering the need to set aside time to visit a healthcare provider for health testing, as expressed by a male student from the FEM: “It’s more because of laziness” (B-FEM). Users reported being fearful of learning their HIV status, with some indicating that they did not feel ready to undergo testing, describing the decision to get tested as particularly difficult to make.

Users further expressed concerns about the confidentiality of the counseling and testing process, and reported a fear of judgment and stigmatization following testing, as illustrated by a female student from the FHS: “What if I go there and then they expose my situation? If I leave there, other colleagues might look at me like [she is] coming out of there, [she] went to get tested’ (laughs)” (G-FHS). Users also demonstrated anticipated regret in relation to a potential HIV diagnosis, reporting a fear of experiencing guilt upon learning of a positive HIV status and the prospect of lifelong treatment. Users acknowledged that such psychological anticipation could lead to adverse mental health outcomes, described by a female student from the FHS.

“I think that most young people do not do health tests, especially HIV tests, because—let us say—most people, like me, look at themselves and think, ‘I’m very healthy, nothing is going on.’ Then one day you decide to go and do a test and it comes back positive, and you get psychologically affected because you will have to undergo treatment. So my biggest fear is that before doing the test I was living normally, without any treatment, and everything was fine for me” (B-FHS).

### Improvement and future implementation

#### Expand and make program more inclusive

Some program users recommended the continuation of the program; however, they expressed a desire for it to be extended to encompass all courses and academic levels within each faculty at the Catholic University of Mozambique (UCM). For instance, at the FHS, students recommended that sensitization activities be extended to include students enrolled in nursing, psychology, hospital administration, laboratory sciences, and pharmacy programs, as they perceived that only medical students were adequately reached by all components and activities of the program. Users further indicated that some of their peers remained unaware of all program components, particularly those related to sensitization for STI and HIV prevention and the provision of VCT services, as indicated a male student from the FHS.

“Yes, I would say that the faculty should continue more and more with this. I think that regarding these activists from the PABODZI group—yes, yes—it should be expanded a bit more, because I strongly believe that there are some students who do not know this group. So it should expand more. I think that in nursing—I do not know if I’m mistaken, but in nursing it seems that this group is a bit more focused on medicine…” (B-FHS).

Program users also recommended expanding the program to include other universities as well. Users expressed the belief that young adults at other universities would equally benefit from the program and could consequently improve their sexual and reproductive health behaviors. Additionally, participants indicated that they would appreciate the incorporation of a religious dimension into the sensitization sessions, given that the program operates within a Catholic institutional context, as suggested by a male student from the FHS: “since this is a Catholic university, there could be a little bit of a religious approach” (B-FHS).

#### Improve delivery of the program

Most program users shared recommendations regarding the behavior change methods and their practical applications needed to improve and adapt the program for future implementation among university students at the UCM and in other settings. They indicated that life skills lectures and sensitization sessions should be made more persuasive, as stated by a male student from the FHS: “I think that the lectures, the PABODZI sessions, should be more persuasive” (B-FHS). It was further suggested that persuasive messages should place greater emphasis on the benefits of getting tested by adopting a gain-framed messaging approach, as participants perceived that some of their peers did not attend or withdrew early from sensitization activities, citing the view that the messages and their delivery were insufficiently motivating. This was illustrated by a male student from the FEM: “so there are many dropouts; they should find another way to encourage students to attend these lectures” (B-FEM).

Program users also recommended the use of an indirect messaging approach, aimed at steering students toward STI and HIV preventive behaviors without addressing the topic directly. For instance, sensitization activities could incorporate a religious framework by shifting the focus away from disease and instead emphasizing being monogamous as a divine commandment, thereby creating a conceptual bridge between faith-based values and the prevention of STIs, including HIV, as stated by a male student from the FHS: “I cannot do what my God does not accept, because there would be no chance of betrayal, in the case of infidelity” (B-FHS).

Furthermore, users indicated that, in society, women are often blamed for STI and HIV transmission, and thus recommended the program no use exclusively female characters. They suggest that the program ensure equal representation of both female and male models in theatrical presentations used for sensitization activities. Participants noted that the predominant use of female role models may have led students to perceive women as the sole transmitters of STIs and HIV, as expressed by a female student from the FEM: “the problem is that they made it seem as if only women transmit these diseases….” (G-FEM).

Some program users further highlighted the need for broader dissemination of information on the benefits of regular VCT service utilization. While they acknowledged that students may already have some awareness of the availability of services at the faculty or other venues, they emphasized the need for more frequent exposure to this information and sustained reinforcement regarding the importance of regular testing and healthcare visits for counseling on health concerns, as stated by a male student from the FHS: “Because many people do not go not because of lack of information, but I think if they encourage them more, two or three times, pushing a bit more, I think adherence would also increase” (B-FHS).

Others program users further recommended that sensitization activities conducted in classrooms and life skills lectures be complemented by general assemblies bringing together students from all courses and academic levels, during which the program coordinator or another designated representative would deliver a discourse addressing STIs, HIV, and other health-related issues, alongside practical activities related to SRH with a particular focus on the use and benefits of VCT services in the prevention of health problems. This was illustrated by a female student from the FEM: “Maybe the university could, once in a while, hold a general talk, not just like the TIRITESSE group going from class to class but, a general talk with all students at the university, from first year to fourth year (uh-huh), and then maybe have things like testing available, more activities to do, maybe some practical things with the students” (G-FEM). It was further suggested that such gatherings could be organized during designated awareness weeks or months, during which the university could simultaneously host counseling and testing campaigns targeting the entire academic community, as suggested a male student from the FEM.

“That there should be a campaign encouraging people to go and get tested, because people know that tests are done there, but they think, ‘one day I’ll go, okay.’ But if it were said, like, ‘Okay, this week,’ or ‘this month, come and get tested because it’s a special month,’ something like that could make people go there” (B-FEM).

The majority of program users also recognized the benefits of utilizing randomly assigned small groups for structured debates. They argued that the random assignment of students to debate groups would ensure heterogeneity in group composition, minimize the risk of negative peer influence, agitation, and coercion, and foster open discussion, given that participants would not necessarily be acquainted with one another. Participants further noted that small group debates are conducive to active participation, as the setting allows all attendees to contribute comfortably. In such a context, participants are more likely to feel at ease sharing their ideas and personal perspectives.

Users described the use of small group debates to promote STI and HIV prevention as a more effective approach than conventional classroom lessons, on the grounds that classroom instruction tends to prioritize the coverage of planned content, with students engaging primarily to pass examinations. In contrast, participants attending small group debates do so with the awareness that they will acquire knowledge in an open and contextually relevant environment, as expressed by a female student from the FEM: “In a class you just want to cover the content, just study it to pass and then move on, whereas in a debate it’s different, you go there aware that you are going to learn… (uh-huh), that you are going to hear things that can have an effect on your life” (G-FEM).

Program users also expressed the need for more skilled peer activists to deliver sensitization activities, recommending that activists receive comprehensive training prior to being assigned to sensitization tasks. It was further suggested that well-trained activists should each be responsible for facilitating two or three groups in the conduct of debates and the delivery of other sensitization activities, as recommended by a female student from the FHS: “I think that first they should take those who are already more involved in the group, for example those from PABODZI, and train them or prepare them better, with each one being responsible for two to three groups” (G-FHS).

#### Timing, duration and frequency

Almost all program users indicated that sensitization activity sessions should be scheduled outside of regular lecture time in order to ensure sufficient time for interaction among attendees, and that activities should be conducted more frequently, as stated by a male student from the FEM: “I would suggest increasing the number of days for the talks” (B-FEM). Users noted that teachers assigned to lectures typically allocate limited time for peer activists to deliver the program content. Extending the duration of sensitization sessions would therefore provide an opportunity for more thorough and inclusive discussion among participants. Users further perceived that increasing the frequency of sensitization activities would enhance the overall effectiveness of the program and serve to motivate and encourage students to engage with VCT services, as indicated a female student from the FHS.

“I think the intervention of the PABODZI group in the classrooms needs to be improved [right], because the group’s interaction has been very shortened. They come at a time when we are already in class and then the lecturers want to come in, so there is not much time for us to interact in a more open way and so on. So we end up being very restricted, and there should be some consideration regarding the time allocated to the PABODZI group in the classrooms” (G-FHS).

#### Diversification

Program users additionally advocated for the diversification of sensitization activities, as well as of the approaches and methods of behavior change used to deliver the program content. They noted that the characters portrayed in theatrical presentations should represent a wide range of student profiles, encompassing both outgoing and reserved students, as the exclusive use of outgoing characters may lead other students to feel that the program is not directed at them and that the issues being addressed are not relevant to their own health behaviors, thereby missing the opportunity to reach all students through STI prevention sensitization. Users further noted that the approaches currently used to deliver sensitization activities are largely limited to classroom visits; however, they suggested that peer activists could, for instance, deliver sensitization content within the framework of recreational events incorporating games and VCT provision campaigns, held during weekends when students are free from the pressures of academic commitments, as suggested a male student from the FHS.

“Well, I think that in part the PABODZI group is very limited, basically just passing through. But I think they should organize more recreational activities…with more diversity” (B-FHS).

#### Program maintenance

Program users´ expressed a strong desire for all components of the program to be maintained indefinitely, including the structured life skills lessons. They described the program as an invaluable source of support for their lives and overall wellbeing. Users acknowledged that, having left their families and parents behind in their home districts or cities, they had come to regard the program as a vital source of social, academic, and health support. They therefore expressed the hope that the program would continue until they had completed their studies and graduated, as suggested a male student from the FHS.

“I think this program should be the oxygen of the university, you know. Because when we leave home and come into the academic environment of a university like this one, we realize that, depending on the culture, the household someone comes from, or the area where they live, they may not know much about sexual life. They do not really know, they deal with taboos or things like that, they become disoriented and very often think and believe that they have to figure things out on their own, through personal initiative or experience, in order to feel experienced. So if this program were the oxygen, if it were constant” (B-FHS).

## Discussion

This study examined the implementation process of a program promoting STI, including HIV, prevention in Mozambique. The study first focused on describing the program implementation in terms of context. The implementation context was shaped by compliance with public health preventive measures against the COVID-19 pandemic. Program implementers were compelled to adapt both the practical application delivery approach and modalities. Life skills teachers were trained to use online platforms to deliver educational content, including life skills lessons. Peer activists adapted the dose, including frequency and duration. Some sessions were shortened and others rescheduled when government-declared lockdowns were in effect. Health advisors referred and encouraged users to seek VCT services wherever available or to return to the office once face-to-face activities resumed.

In light of the contextual challenges posed by COVID-19 lockdowns, previous studies have similarly reported negative effects on the provision of health services, including VCT worldwide (32–34). For example, a study conducted across 44 countries in Asia, Latin America and the Caribbean, Europe, and Africa found that restrictive measures to prevent COVID-19 significantly reduced HIV testing rates in all countries examined, including Mozambique (32).

Previous implementation studies have also similarly reported adaptations to implementation strategies in response to COVID-19 lockdowns (33, 35–38), aimed at maintaining the provision of VCT and other services, including comprehensive sexuality education and sensitization activities to promote VCT engagement. Reported adaptations included: the use of telehealth (36); video content delivered via social media platforms, alongside social networking tools such as Facebook and WhatsApp (33); telephone and virtual communication, including email (35, 38) and the facilitation of home-based HIV self-testing device distribution (37).

The skills and competence of this program’s implementers likely contributed to the observed results. For instance, not all life skills teachers had training in health or biomedical sciences, and health advisors also reported needing refresher training on VCT service provision. Focus group discussion data further revealed that users recommended skills improvement for peer activists, suggesting perceived gaps in the competence of some members of this implementers group.

These findings underscore an important lesson for researchers and health promoters: when designing and planning programs, alternative implementation strategies should always be considered and anticipated to mitigate the potential influence of contextual factors. This is particularly critical in resource-limited settings, where bureaucratic constraints, funding shortfalls, and limited availability of skilled human resources pose persistent challenges. In retrospect, this program could have benefited from adopting additional adaptive strategies across all its components. For example, rather than solely referring students to seek VCT services at external health facilities, the provision of HIV self-testing (HIVST) devices could have served as a viable and more accessible alternative (37). However, this would have required additional funding for the procurement of HIVST devices, ethical approval for their use within the study, and supplementary training for health advisors on how to provide counselling and support in the context of HIV self-testing.

The study also examined program reach, attendance, and fidelity. Overall, all program components were well received by both implementers and users. Interview data indicated that attendance at life skills education sessions was high, with more than half of users reporting attendance at 70–100% of scheduled lessons. Attendance at sensitization sessions was also acceptable, with nearly half of users reporting attendance at 70–100% of scheduled sessions. However, a considerable proportion of users did not report utilizing VCT services, suggesting that a notable number of participants were not reached by all programs´ components.

These findings may be partly explained by the contextual challenges described above, which necessitated adaptations to program delivery modalities. Some adaptation strategies required, for example, additional resources to ensure full attendance at life skills lessons, as well as extra time for students to seek and access VCT services outside the faculty. Focus group discussion data also identified additional factors that may have negatively affected program reach. Specifically, users noted that sensitization activities were not scheduled at convenient times, that is, outside of regular academic lessons. Furthermore, some users indicated that they did not fully understand the primary goal of the program.

These findings are consistent with those of previous implementation studies on sexual and reproductive health, including VCT promotion (7, 15, 16, 32, 36). For instance, one implementation study conducted in South Africa found that approximately 6.6% of participants refused peer support linkage, citing being too busy and not fully understanding the intervention (16). Furthermore, another South African study found that some participants showed limited interest in broader sexual health information, while others were uncomfortable receiving health guidance from individuals not perceived as healthcare professionals (17).

These findings further highlight that it is not sufficient to focus solely on adaptation strategies and contingency planning to address contextual challenges during program implementation. Equal consideration should be given to implementer readiness, that is, how prepared implementers are to adopt new delivery approaches and modalities. Equally important, contingency planning should incorporate strategies to equip users with the tools and resources necessary for full engagement with the adapted implementation process.

Interview data from users revealed that implementation fidelity was not fully maintained across all program components. Regarding life skills lessons, the planned structure required that each topic begin with an open student discussion before the lecture and conclude with a content summary. However, participants indicated that not all lessons followed this planned structure. Similarly, sensitization activities were not always delivered as intended; users reported that not all sessions began with a theatrical play or opening questions designed to stimulate participant engagement. Furthermore, in VCT consultations, less than half of users reported that they had discussed a health calendar for the regular use of VCT services with their health advisor. Failure to maintain implementation fidelity, specifically, adherence to program guidelines has been reported in previous implementation studies (15, 39).

These results may be attributed not only to the contextual challenges described above, but also to other factors, such as the degree of program integration into the existing curricular framework and implementers’ prior experience with SRH promotion. Teacher training and teaching experience, not only with the subject matter, but also with interactive teaching methods, might have affected implementation fidelity (2). In this study, for example, some life skills teachers had fewer than two years of teaching experience, suggesting that program managers should consider assigning more experienced teachers and ensure regular refresh training to all groups of implementers.

Finally, this study explored the experiences and barriers that students encountered in accessing VCT services, as well as their recommendations for program improvement and future implementation. Users indicated that initial exposure to the STI and HIV prevention program within the university setting elicited emotional responses; however, over time, they normalized the topic and became more comfortable discussing sexual and reproductive health issues. Users generally found the program interesting and reported a positive impact on their lives. Nevertheless, certain challenges persisted in hindering their engagement with VCT services, prompting users to recommend improvements for future program implementation. These recommendations included: expanding the program and making it more inclusive; improving the overall delivery of the program; reviewing the timing, duration, and frequency of activities; and diversifying the content of awareness sessions.

These results are consistent with those of previous implementation studies (40–43), although most of these studies focused on assessing the perspectives of adolescents and young people exposed to sexual and reproductive health promotion programs more broadly. For instance, a systematic review consolidating available evidence on the perceptions, experiences, and needs of young people regarding school-based sexual health education concluded that adolescents had mixed reactions toward such programs and expressed a need for greater inclusivity (43). A safe learning environment, access to well-trained educators, and the use of interactive teaching approaches are consistently recommended by participants (43). Consequently, future programs need to incorporate diversified sexual health behavior outcomes and adopt a multidisciplinary delivery approach, engaging a combination of teachers, health professionals, and peers (43).

### Strengths

The study applied a mixed-methods approach combining qualitative and quantitative data collection strategies, which yielded reliable and consistent results. Additionally, data were collected from multiple sources using different instruments and techniques (triangulation) to assess various domains of implementation outcomes, producing comprehensive and inclusive results.

### Limitations

The results may have been influenced by the following limitations. (1) social desirability bias may have affected the results, as program users might have felt compelled to evaluate their teachers and peer activists positively out of gratitude. (2) given the sensitive nature of the topic, STI and HIV prevention, users may have provided socially desirable rather than accurate responses. (3) collecting data from users at the end of the intervention may have introduced recall bias, owing to memory limitations associated with current emotions, beliefs, and the time elapsed since earlier program activities. These limitations were mitigated through methodological triangulation, prolonged engagement with the program, comprehensive sampling strategies, and measures to ensure the impartiality of the research team.

## Conclusion

The results of this study contribute meaningful and updated information to the literature on the promotion of Voluntary Counselling and Testing (VCT) services in sub-Saharan Africa, particularly in pandemic contexts where face-to-face provision of social services, including education and health care, is restricted. These findings may support researchers, guide stakeholders, and inform public health policymakers on the future development and implementation, as well as, the adaptation of existing STIs and HIV prevention programs, including the promotion of VCT utilization among university students in Mozambique and similar settings.

Despite the challenges identified across the assessed implementation domains: context, reach, dose and fidelity; the program at the Catholic University of Mozambique is well established and integrated into the curricular framework across all academic courses. Nevertheless, opportunities for improvement should be actively pursued to accommodate the evolving needs and expectations of students, address implementation gaps, and ensure a meaningful impact on health behavior outcomes, particularly the uptake of VCT services.

## Data Availability

The raw data supporting the conclusions of this article will be made available by the authors, without undue reservation.
